# An updated systematic review and meta-analysis of the efficacy and safety of early oral feeding vs. traditional oral feeding after gastric cancer surgery

**DOI:** 10.3389/fonc.2024.1390065

**Published:** 2024-09-04

**Authors:** Dong Xu, Junping Li, Jinchao Liu, Pingjiang Wang, Jianjian Dou

**Affiliations:** ^1^ Department of General Surgery, Zibo Municipal Hospital, Zibo, Shandong, China; ^2^ Department of Oncology, Zibo Municipal Hospital, Zibo, Shandong, China; ^3^ Department of Radiation, Zibo Municipal Hospital, Zibo, Shandong, China

**Keywords:** early oral feeding, traditional oral feeding, gastric cancer, gastrectomy, meta-analysis

## Abstract

**Introduction:**

Early oral feeding (EOF) has been shown to improve postoperative recovery for many surgeries. However, surgeons are still skeptical about EOF after gastric cancer surgery due to possible side effects. This updated systematic review and meta-analysis aimed to investigate the efficacy and safety of EOF in patients after gastric cancer surgery.

**Methods:**

Randomized controlled trials (RCTs) investigating EOF in patients after gastric cancer surgery were searched in the databases of PubMed, Embase, Clinicaltrials.gov, and Cochrane from 2005 to 2023, and an updated meta-analysis was performed using RevMan 5.4 software.

**Results:**

The results of 11 RCTs involving 1,352 patients were included and scrutinized in this analysis. Hospital days [weighted mean difference (WMD), −1.72; 95% confidence interval (CI), −2.14 to −1.30; p<0.00001), the time to first flatus (WMD, −0.72; 95% CI, −0.99 to −0.46; p<0.00001), and hospital costs (WMD, −3.78; 95% CI, −4.50 to −3.05; p<0.00001) were significantly decreased in the EOF group. Oral feeding tolerance [risk ratio (RR), 1.00; 95% CI, 0.95–1.04; p=0.85), readmission rates (RR, 1.28; 95% CI, 0.50–3.28; p=0.61), postoperative complications (RR, 1.02; 95% CI, 0.81–1.29; p=0.84), anastomotic leakage (RR, 0.83; 95% CI, 0.25–2.78; p=0.76), and pulmonary infection (RR, 0.65; 95% CI, 0.31–1.39; p=0.27) were not significantly statistical between two groups.

**Conclusion:**

This meta-analysis reveals that EOF could reduce hospital days, the time to first flatus, and hospital costs, but it was not associated with oral feeding tolerance, readmission rates, or postoperative complications especially anastomotic leakage and pulmonary infection, regardless of whether laparoscopic or open surgery, partial or total gastrectomy, or the timing of EOF initiation.

## Introduction

Gastric cancer (GC) constitutes a significant global health challenge, characterized by a poor prognosis (1). It ranks fifth in incidence among malignancies and fourth in cancer-related mortality worldwide (1). Surgical intervention remains the primary therapeutic approach for gastric cancer (2). Subtotal and total gastrectomy represent the most frequently employed surgical techniques in the management of gastric cancer patients (3). Nevertheless, malnutrition frequently afflicts individuals diagnosed with gastric cancer. Malnutrition in gastric cancer patients significantly elevates the risk of postoperative complications and adversely affects overall survival rates following gastrectomy (4). Consequently, ensuring adequate nutritional support is paramount for enhancing postoperative outcomes in gastric cancer patients.

In order to accelerate the postoperative recovery of patients after gastrointestinal surgery, the concept of fast track surgery (FTS) or enhanced recovery after surgery (ERAS) has been proposed and applied in a variety of operations (5, 6), such as colorectal cancer (7), lung cancer (8), liver cancer (9), and gynecological surgery (10). Early oral feeding (EOF) is widely recognized as a critical component of FTS and ERAS protocols and has demonstrated favorable outcomes following gynecological tumor surgery and colorectal procedures (11, 12). Despite evidence from several studies indicating the safety and efficacy of EOF post-gastrectomy (13–15), clinical surgeons remain cautious about its implementation in gastric cancer surgery, citing potential adverse effects such as anastomotic leakage, oral feeding intolerance, and aspiration pneumonia.

To further elucidate the efficacy and safety of EOF, we performed a comprehensive and updated systematic review and meta-analysis of randomized controlled trials (RCTs), aimed at offering evidence-based guidance for clinicians. Additionally, we examined the impact of various surgical approaches (laparoscopy versus open surgery, total gastrectomy versus subtotal gastrectomy) and the timing of early feeding on the outcomes of EOF in postoperative gastric cancer patients.

## Methods

### Search strategy

The relevant RCTs in which language was restricted to English from 2005 to 2023 were searched from PubMed, Embase, Clinicaltrials.gov, and Cochrane. Search terms include “early oral feeding or early oral intake,” “gastric cancer,” “gastrectomy”, “fast track surgery,” and “enhanced recovery after surgery.” Only RCTs were included in the meta-analysis after all studies were browsed by two independent reviewers.

### Criteria for selection

The inclusion criteria were as follows: (1) RCTs, (2) adult patients (≥18 years old), (3) gastric cancer patients receiving oral feeding after gastrectomy, and (4) data on the outcomes related to hospital days, the time to first flatus, hospital costs, oral feeding tolerance, readmission rates, postoperative complications, anastomotic leakage, and pulmonary infection.

The exclusion criteria were as follows: animal trials, non-RCTs, comments, case reports, letters, ongoing trials, protocols, and studies lacking of applicable data.

### Data extraction

Two researchers extracted the following data independently: first author, year, country, trial design, surgical approach, sample size, time of oral feeding, age, and gender (m/f). The outcomes analyzed in the meta-analysis included (1) hospital days, (2) the time to first flatus, (3) hospital costs, (4) oral feeding tolerance, (5) postoperative complications, (6) readmission rates, (7) anastomotic leakage, and (8) pulmonary infection. Two sets of data were collected when there were two operation methods within the same RCTs.

### Quality assessment

The quality of the retrieved RCTs was assessed through the Cochrane risk of bias tool (16). The quality items were random sequence generation, allocation concealment, blinding of participants and personnel, blinding of outcome assessment, incomplete outcome data, selective outcome reporting, and other bias. Any uncertainties about the quality assessment were resolved through the discussion among all reviewers.

### Data analysis

Review Manager version 5.4 was used to analyze the data included in the meta-analysis. The continuous data of the included RCTs were elucidated using weighted mean difference (WMD) along with the corresponding 95% confidence interval (CI). The dichotomous data of the included RCTs were performed by using the risk ratio (RR). Estimation of the sample mean and standard deviation from the sample median and range was accomplished according previously published methods (17, 18). The result of meta-analysis was statistical significant when p-value < 0.05. The heterogeneity in data analysis was analyzed by using I^2^ statistic. A fixed effect model would be used for results where the I^2^ value is <50% and has insignificant heterogeneity. Otherwise, the random effect model was applied.

### Subgroup analysis, sensitivity analysis, and publication bias

Subgroup analyses were performed according to the pre-specified factors: operation methods (laparoscopy vs. open surgery, total gastrectomy vs. subtotal gastrectomy) and early feeding time on EOF of patients with gastric cancer after operation. A sensitivity analysis was performed by excluding trials recruiting participants with particular conditions or trials with characteristics that were different from those in other trials. Publication bias was assessed with funnel plots and Egger’s and Begg’s tests. A p-value < 0.05 was considered statistically significant.

## Results

### Characteristics of the individual studies

**Figure 1:**
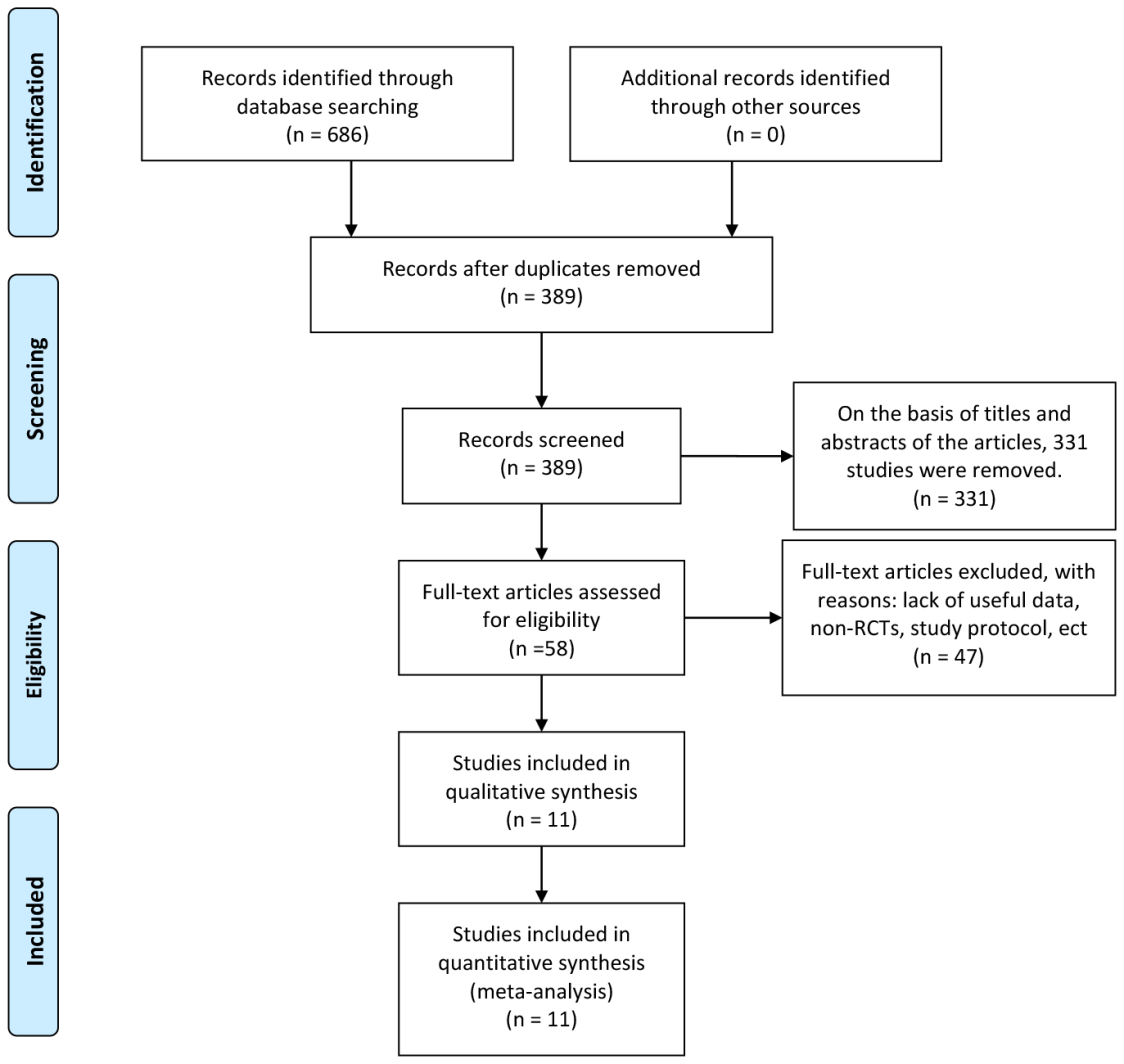
Flowchart of study inclusion.

A total of 686 articles were identified according to the searching terms from each database. Following a preliminary screening of titles and abstracts, 331 trials were excluded. A total of 47 articles were exclude due to lack of useful data, non-RCTs, study protocol, etc. Finally, 11 RCTs (19–29) encompassing 1,352 participants (EOF group: 671 participants vs. traditional oral feeding (TOF) group: 681 participants) were included in our meta-analysis (**Figure 1**). The characteristics of the 11 selected RCTs are revealed in **Table 1**.

**Table 1:** General characteristics of the studies included.

Study, year, country	Trial design	Surgical approach	Sample size		Time of oral feeding		Age	Gender (m/f)
			EOF	DOF	EOF	DOF		
Liu et al., 2010 (19), China	RCT	Open gastrectomy, with lymph node dissection	33	30	Day of surgery: Oral intake clear fluid ≈50 mL POD1: Semiliquid diet 50–100 mL POD2: Semiliquid diet 100–200 mL POD3: Semiliquid diet 200–400 mL POD4: Semiliquid diet POD5: Solid diet	Diet reintroduced in same manner as in optimized group on PODs 1, 2, and 3 after bowel venting. POD5: Semiliquid diet POD5+: Solid diet	28–81	33/29
Wang et al., 2010 (20), China	RCT	Open distal or proximal subtotal resection or total gastrectomy, standard D2 total gastrectomy	45	47	Day of surgery: a little clear water POD1: 0.5 L liquid (follow a stepwise plan from water to other liquids to semi-fluids to normal food; adhere to the premise of eating little and often) POD2: 1 L liquid+others as above POD3: Continue as above	Oral intake is initiated if normal bowel sounds are heard (follow a stepwise plan from water to other liquids to semi-fluids to normal food; adhere to the premise of eating little and often)	<80	61/31
Hur et al., 2011 (21), Korea	RCT	Open subtotal gastrectomy or total gastrectomy	28	26	POD1: sips of water POD2: liquid diet POD3: a soft diet	POD3: sips of water POD4–5: liquid diet POD6: a soft diet	<65 or >=65	33/21
Chen Hu et al., 2012 (22), China	RCT	laparoscopy-assisted radical distal gastrectomy or open distal gastrectomy	40	42	6–8h after surgery, following a stepwise program from warm clear water to carbohydrate drink to TPF, then to semi-fluids to normal food.	After the first flatus, the patient progressed from fluids to semi-fluids and then to normal food.	25–75	41/41
Kim et al., 2012 (23), Korea	RCT	laparoscopic distal gastrectomy with intracorporeal anastomosis (Billroth I, Billroth II, or Roux-en-Y type)	22	22	SOW were started 48 h after surgery POD2: Clear liquid diet at dinner POD3: Clear liquid diet at breakfast, full liquid diet at lunch and dinner POD4: Soft diet at breakfast and lunch	SOW after flatus, diet build-up; three steps (clear liquid–full liquid–soft diet), one step a day, from the day after start day of SOW	Unclear, mean 55.05	
Feng et al., 2013 (24), China	RCT	Standard laparotomy approach, standard D2 total gastrectomy	59	60	Day of surgery: 500–1,000 mL glucose saline. POD1: 2,000–3,000 mL liquid food containing 1,000–1,200 kcal per day	Oral intake initiated after flatus (following a stepwise plan from water to other liquids to semi-fluids to normal food)	28–75	85/34
Hong et al., 2014 (25), China	RCT	laparoscopic distal gastrectomy	40	44	POD1–2: a clear liquid diet POD3: a soft diet	POD 3–4: clear liquid diet; POD 5: soft diet.	18–75	59/25
Li et al., 2015 (26), China	RCT	Open radical gastrectomy	150	150	POD1: a small amount of drinking water. POD2: 500 mL of fractionated oral enteral nutrition POD3: 1,000 mL of oral Jevity was given multiple times in addition to a small amount of liquid diet	After anal exhaust, the patient began to drink water orally. If no discomfort, the intake of water and liquid and semi-liquid diets were gradually increased.	Unclear, mean 59.8	154/146
Shimizu et al., 2018 (27), Japan	RCT	distal or total gastrectomy either laparoscopically or via laparotomy	102	114	POD1–3: a diet of iEAT^®^ POD 4: ordinary hospital diets	conventional nutritional management	20–80	137/79
Wang et al., 2019 (28), China	RCT	total laparoscopic radical gastrectomy, standard D2 lymphadenectomy, and the main digestive tract anastomoses	51	49	POD1: liquid food on the morning POD2–6: liquid foods and semi-liquids were adopted according to the different tolerances of different individuals	POD4–6: liquid food POD7: the same oral intake as the EOF group	18–75	71/29
Gao et al., 2019 (29), China	RCT	laparoscopic distal gastrectomy or laparoscopic radical gastrectomy	101	97	POD2: the oral fluid diet POD3: Semi-liquid food and soft food Insufficient intake of oral nutrition was supplemented by intravenous fluids.	Oral intake initiated after flatus (following a stepwise plan from water to other liquids to semi-fluids to normal food)	44–80	123/75

EOF, early oral feeding; TOF, traditional oral feeding; POD, postoperative day; SOW, sips of water; iEAT^®^, a commercially available food (EN Otsuka Pharmaceutical Co., Ltd., Hanamaki, Japan).

### Quality of the RCTs

**Figure 2:**
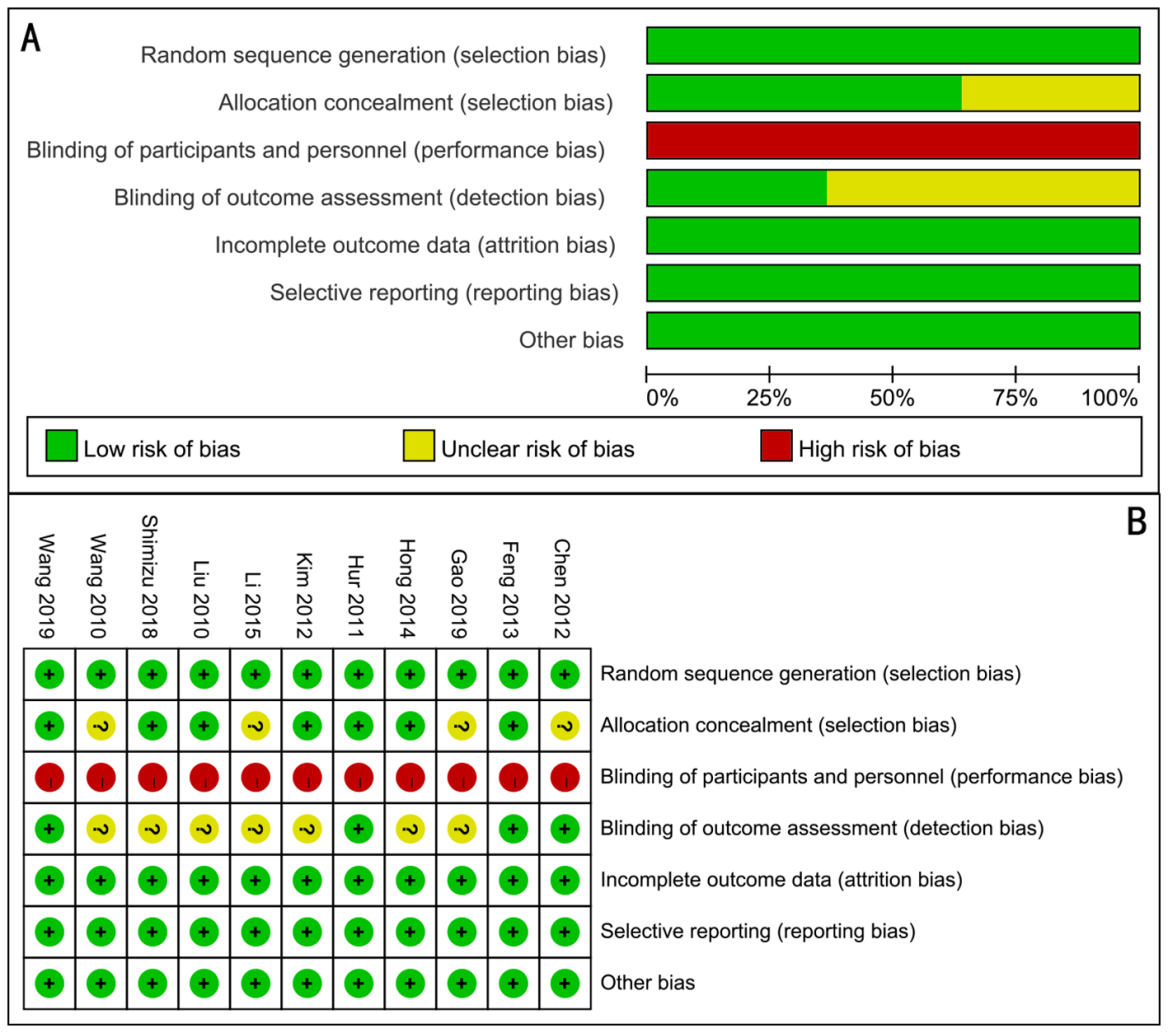
Assessment of randomized study quality. **(A)** Risk of bias graph, **(B)** risk of bias summary.

All of the 11 articles included in the meta-analysis were RCTs. The assessment of risk of bias of studies is shown in **Figure 2**. It was difficult to ensure blinding because the oral feeding was easy to distinguish.

## Result of meta-analysis

### Hospital days

**Figure 3:**
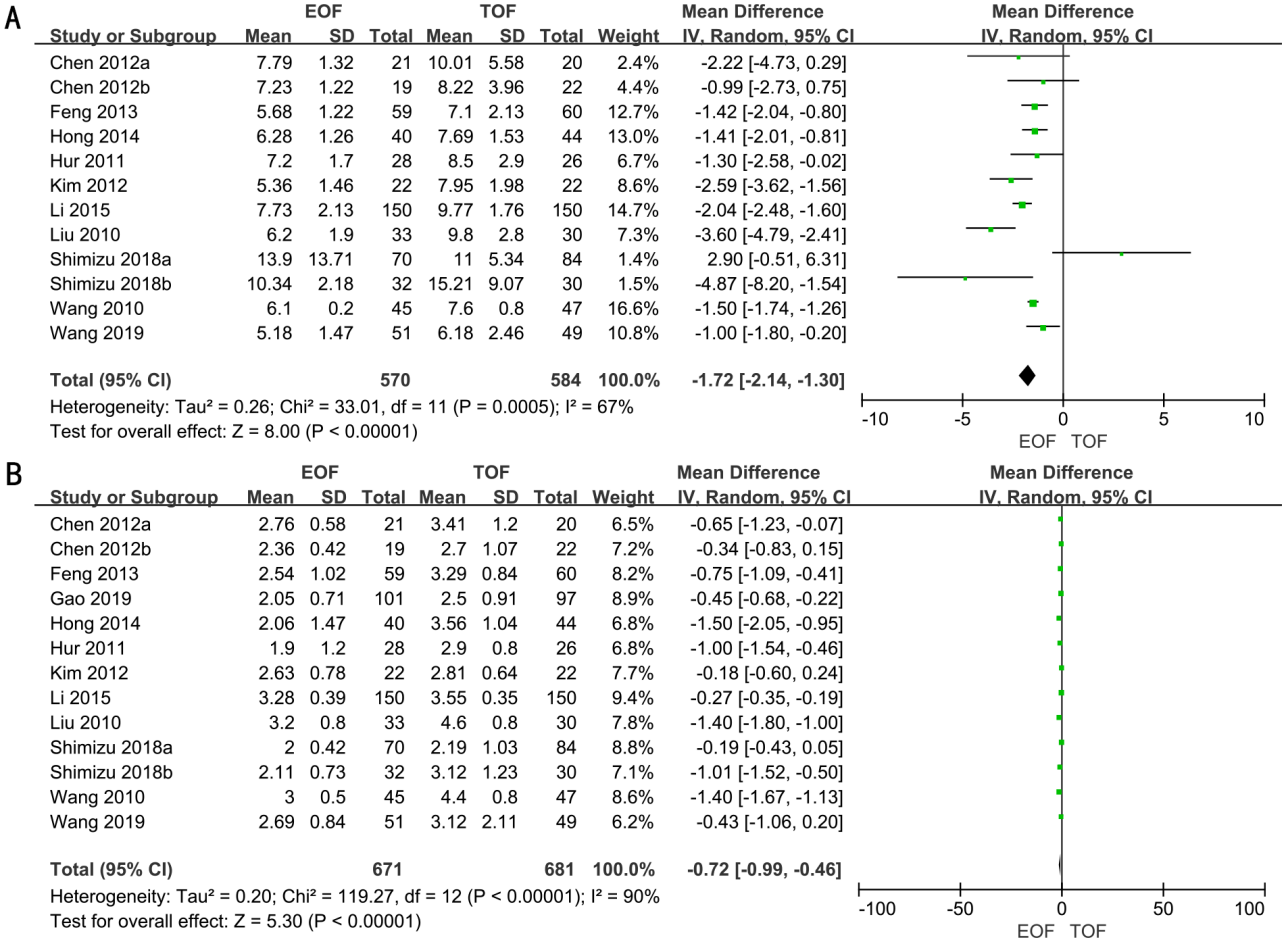
Forest plots of hospital day **(A)** and the time to first flatus **(B)**.

A total of 10 RCTs encompassing a total of 1,154 patients were analyzed to assess the length of hospital stay. A random-effects model was employed to evaluate the difference in hospital day duration between the two groups. The forest plot illustrated a significant reduction in hospital stay duration for the EOF group compared to the TOF group (WMD, −1.72; 95% CI, −2.14 to −1.30; p<0.00001) (**Figure 3A**).

Subgroup analyses revealed that patients in the EOF group experienced significantly shorter hospital stays compared to the TOF group, irrespective of surgical approach (laparoscopic vs. open surgery), extent of gastrectomy (partial vs. total), or the timing of EOF initiation (**Supplementary Figure 1**).

### The time to first flatus

A total of 11 RCTs, involving 1,352 patients, were adopted to analyze the time to first flatus. Employing a random-effects model, we assessed the difference in time to first flatus between two groups. The result revealed that the time to first flatus was significantly shorter in the EOF groups (WMD, −0.72; 95% CI, −0.99 to −0.46; p<0.00001) (**Figure 3B**).

Subgroup analyses indicated that patients in the EOF group experienced a significantly shorter time to first flatus compared to the TOF group, regardless of the type of surgery (laparoscopic vs. open), the extent of gastrectomy (partial vs. total), or the timing of EOF initiation (**Supplementary Figure 2**).

### Hospital costs

**Figure 4:**
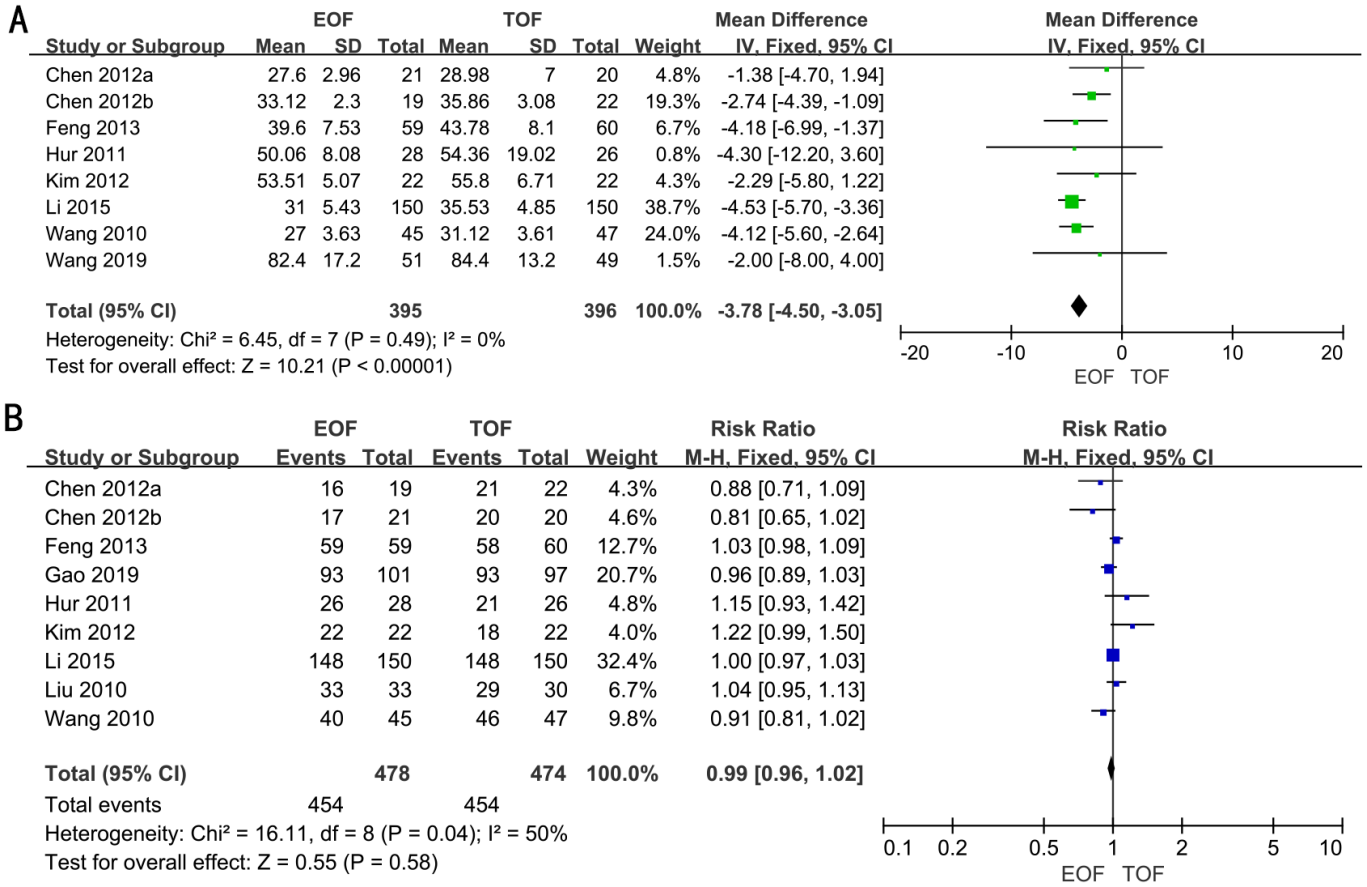
Forest plots of hospital costs **(A)** and oral feeding tolerance **(B)**.

Seven RCTs full of 791 patients were applied to assess the hospital costs. We adopted a fixed-effects model to evaluate the hospital costs between two groups (WMD, −3.78; 95% CI, −4.50 to −3.05; p<0.00001). The forest plot revealed a significant decrease in hospital costs within the EOF group compared to the TOF group (**Figure 4A**).

Subgroup analyses demonstrated that patients in the EOF group incurred lower hospital costs compared to those in the TOF group, irrespective of surgical method (laparoscopic vs. open), extent of gastrectomy (partial vs. total), or the timing of EOF initiation (**Supplementary Figure 3**).

### Oral feeding tolerance

Eight RCTs full of 952 patients were adopted to evaluate oral feeding tolerance. A random-effects model was used to assess oral feeding tolerance between two groups [risk ratio (RR), 1.00; 95% CI, 0.95–1.04; p=0.85). The results revealed that there was no significant difference in oral feeding tolerance between the two groups (**Figure 4B**).

Subgroup analyses revealed that patients in both groups exhibited similar oral feeding tolerance, irrespective of the surgical approach (laparoscopic vs. open), the extent of gastrectomy (partial vs. total), or the timing of EOF initiation (**Supplementary Figure 4**).

### Postoperative complications

**Figure 5:**
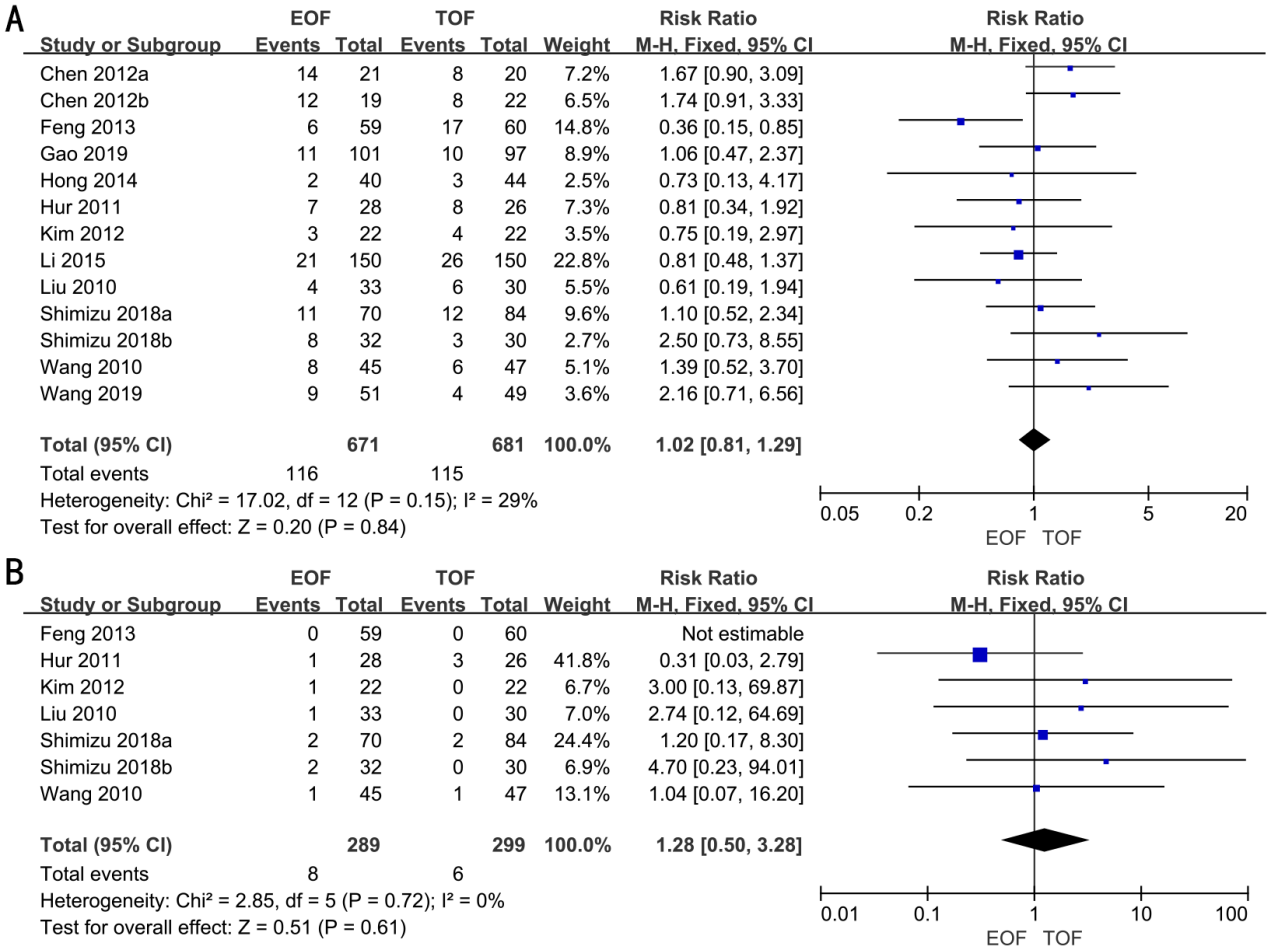
Forest plots of postoperative complications **(A)** and readmission rates **(B)**.

In 11 RCTs, enrolling 1,352 patients, the rate of postoperative complications was analyzed. We adopted a fixed-effects model. The forest plot revealed that the improvements in the rate of postoperative complications were similar between the two groups (RR, 1.02; 95% CI, 0.81–1.29; p=0.84) (**Figure 5A**).

Subgroup analyses indicated that there was no significant difference in the rate of postoperative complications between the groups, irrespective of surgical approach (laparoscopic vs. open), extent of gastrectomy (partial vs. total), or timing of EOF initiation (**Supplementary Figure 5**).

### Readmission rates

Six RCTs full of 588 patients were applied to assess the readmission rates. A fixed-effects model was adopted. The result revealed that there was no significant difference in the readmission rates in both groups (RR, 1.28; 95% CI, 0.50–3.28; p=0.61) (**Figure 5B**).

Subgroup analyses revealed that readmission rates were similar between the two groups, regardless of the type of surgery (laparoscopic vs. open), the extent of gastrectomy (partial vs. total), or the timing of EOF initiation (**Supplementary Figure 6**).

### Anastomotic leakage

**Figure 6:**
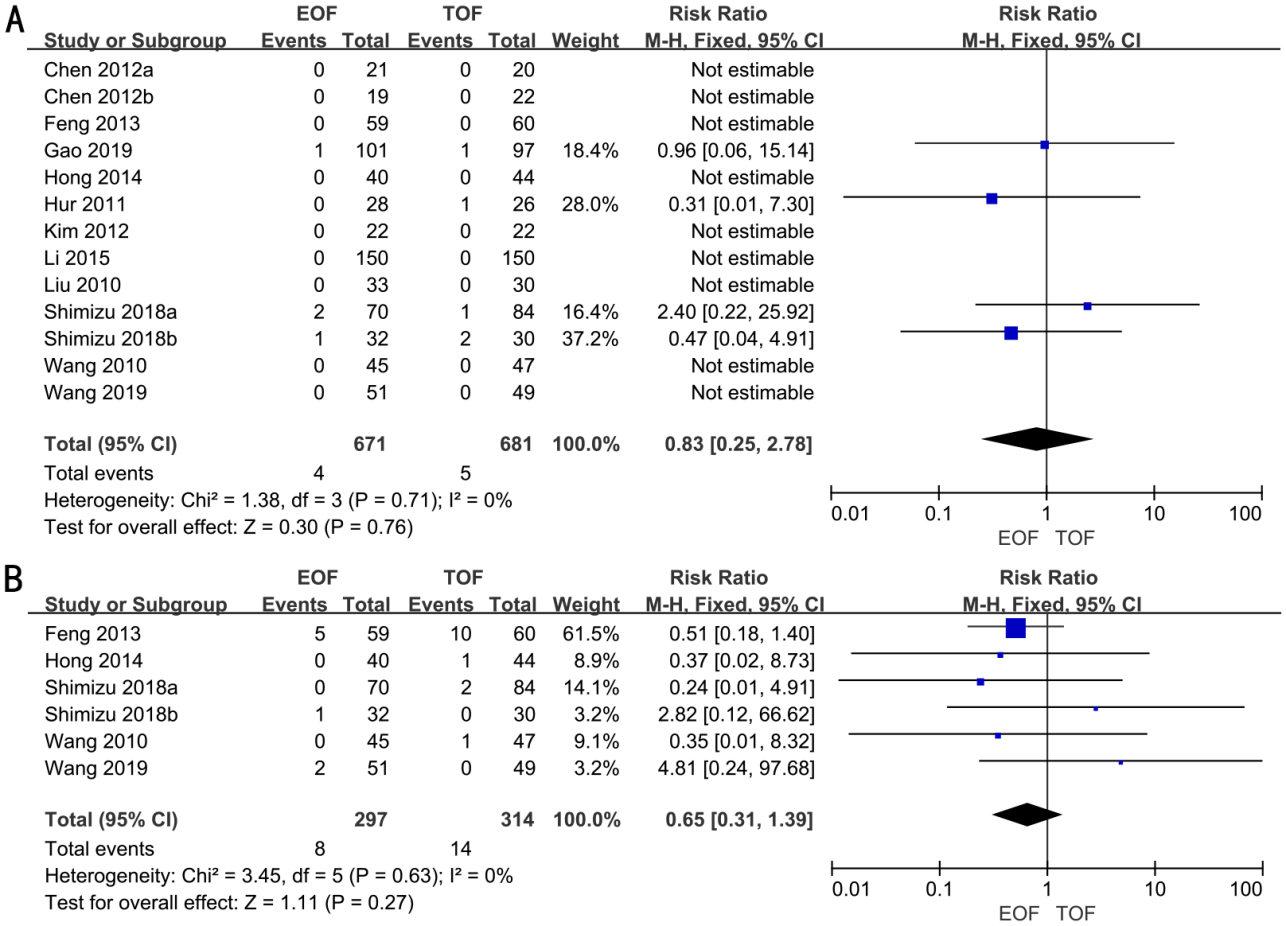
Forest plots of anastomotic leakage **(A)** and pulmonary infection **(B)**.

In 11 RCTs, enrolling 1,352 patients, the rate of anastomotic leakage was analyzed. We adopted a fixed-effects model. The forest plot revealed that the improvements in the rates of anastomotic leakage were similar between the two groups (RR, 0.83; 95% CI, 0.25–2.78; p=0.76) (**Figure 6A**).

Subgroup analyses indicated that there was no significant difference in the rates of anastomotic leakage between the groups, irrespective of surgical approach (laparoscopic vs. open), extent of gastrectomy (partial vs. total), or timing of EOF initiation (**Supplementary Figure 7**).

### Pulmonary infection

Five RCTs full of 611 patients were applied to assess the rate of pulmonary infection. A fixed-effects model was adopted. The result revealed that there was no significant difference in the rates of pulmonary infection in both groups (RR, 0.65; 95% CI, 0.31–1.39; p=0.27) (**Figure 6B**).

Subgroup analyses indicated that the rates of pulmonary infection were similar between the two groups, irrespective of the surgical approach (laparoscopic vs. open), extent of gastrectomy (partial vs. total), or timing of EOF initiation (**Supplementary Figure 8**).

### Sensitivity analysis

We performed a sensitivity analysis by including only studies with total sample size of at least 50 participants to evaluate the robustness of the outcomes (**Table 2**). The results indicated that the sensitivity analysis did not substantially alter the outcomes. The sensitivity analysis results affirmed the reliability of the evidence presented in this meta-analysis.

**Table 2:** Sensitivity analysis results.

Outcomes	Number of studies	Patients		WMD/RR	95% CI	p*-*value	I^2^
		EOF	DOF				
Hospital days	9	508	520	−1.66	−2.12, −1.19	<0.00001	72
The time to first flatus	9	609	617	−0.82	−1.14, −0.50	<0.00001	92
Hospital costs	5	333	332	−4.31	−5.16, −3.45	<0.00001	0
Oral feeding tolerance	6	416	410	1.00	0.97, 1.02	0.78	36
Postoperative complications	9	609	617	0.92	0.71, 1.21	0.56	20
Readmission rates	6	267	277	1.15	0.43, 3.13	0.78	0
Anastomotic leakage	9	609	617	0.83	0.25, 2.78	0.76	0

EOF, early oral feeding; TOF, traditional oral feeding; WMD, weighted mean difference; RR, risk ratio; CI, confidence interval.

### Publication bias

**Figure 7:**
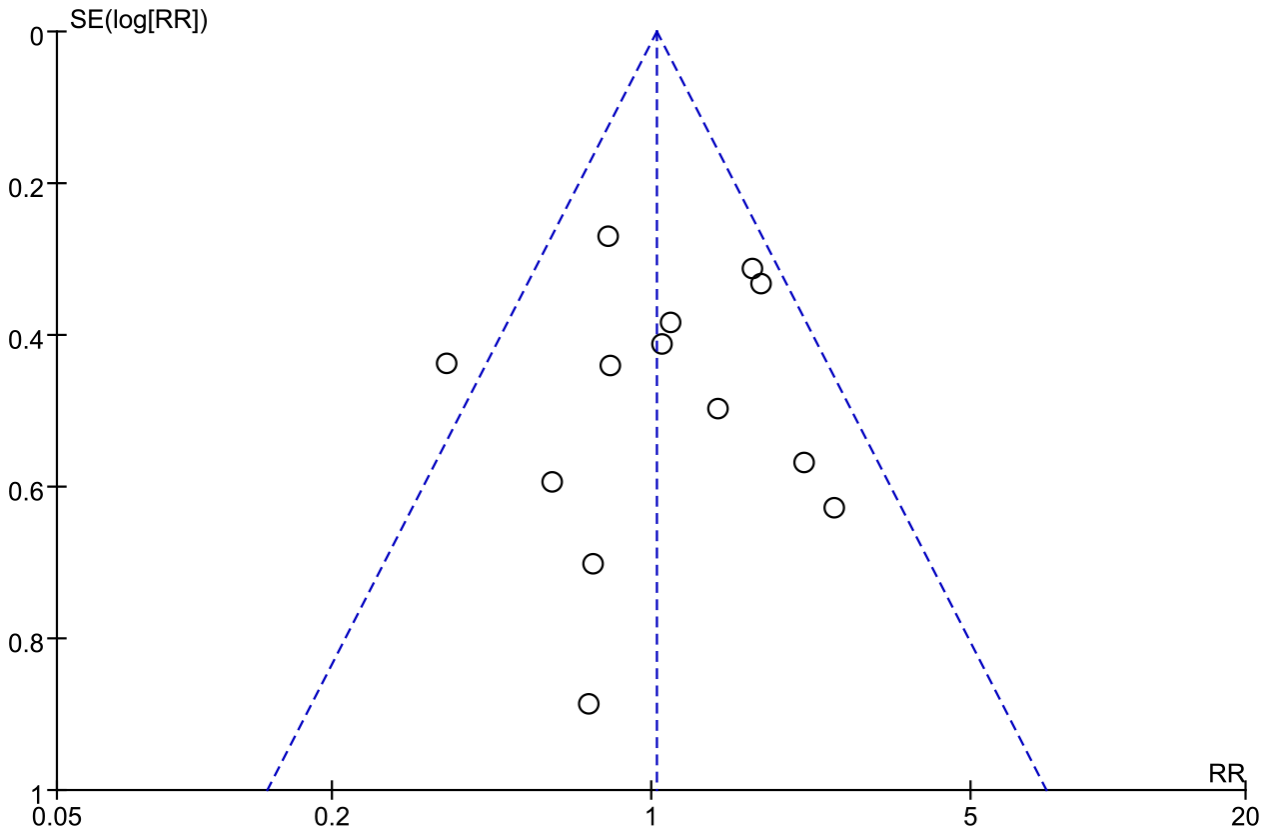
Funnel plot of the studies represented in the meta-analysis. RR, risk ratio; SE, standard error.

A funnel plot was generated to evaluate publication bias in the meta-analysis of postoperative complications. The plot indicated no evidence of publication bias (**Figure 7**). Furthermore, publication bias was not detected in the analyses of “Hospital days,” “Time to first flatus,” “Hospital costs,” “Oral feeding tolerance,” “Readmission rates,” “Anastomotic leakage,” and “Pulmonary infection” (**Supplementary Figure 9**).

## Discussion

The updated systematic review and meta-analysis included 11 RCTs that provided the most recent data on the safety and efficacy of EOF after gastrectomy for gastric cancer. The results indicated that EOF significantly reduced hospital stay duration, expedited time to first flatus, and lowered hospitalization costs. Additionally, EOF did not increase the incidence of oral feeding intolerance, readmission rates, or postoperative complications, including anastomotic leakage and pulmonary infection. Subgroup analyses based on surgical approach (laparoscopic vs. open), extent of gastrectomy (partial vs. total), and timing of EOF initiation, along with sensitivity analyses, corroborated these findings.

Gastrectomy is the definitive treatment for patients with gastric cancer (30), and their nutritional status is also very important. However, malnutrition is prevalent among gastric cancer patients due to tumor-related factors, nausea, vomiting, bleeding, obstruction, surgical trauma, and metabolic disorders, and it is associated with increased postoperative complication rates (31–33). Therefore, nutritional support is particularly important for patients with gastric cancer after surgery. EOF, an essential part of FTS and ERAS, has been suggested to benefit patients with gastric cancer after surgery (34). However, the clinical application of EOF after gastric cancer surgery remains controversial. The main reason is the worry about the incidence of postoperative complications, such as anastomotic leakage, oral feeding intolerance, and aspiration pneumonia.

The present meta-analysis revealed that EOF after gastrectomy for gastric cancer would not increase the incidence of oral feeding intolerance, readmission rates, or postoperative complications, especially anastomotic leakage and pulmonary infection. In the traditional surgical treatment of gastric cancer, patients underwent routine fasting and gastrointestinal decompression for 5–7 days after surgery until exhausted based on the concern that EOF would increase vomiting, flatulence, anastomotic fistula, etc. (29, 35, 36). Nelson reported that gastrointestinal decompression did not reduce the incidence of postoperative complications (37). Even gastrointestinal decompression was found to increase the rate of pulmonary complications and prolong the time to first flatus (38–40). Lu showed that there was no significant difference in the incidence of EOF intolerance, such as abdominal distension and nausea (14). A meta-analysis also reported that feeding intolerance was comparable between EOF and TOF groups (41). For readmission rates, multiple studies and meta-analyses showed that readmission rates were similar between two groups (41–43). Concerning postoperative complications, especially anastomotic leakage and pulmonary infection, in 2004, Suehiro first showed that there was no evidence that EOF would increase postoperative complications and mortality, including anastomotic leakage (44). Jang’s study demonstrated EOF could facilitate early bowel recovery without increasing the incidence of complications, including anastomotic leakage and aspiration pneumonia (45). They also showed a lower incidence of anastomotic leakage in the EOF group; this may be related to improvement in operation techniques, devices, and perioperative care with time (45). A propensity score matching analysis reported that no significant differences were found in the postoperative complications (42). Tadano revealed that EOF was beneficial in the recovery of peristalsis and promoted anastomotic healing in the rat model (46). Another animal study also reported that EOF can promote anastomotic healing and strengthen anastomotic strength of intestinal and somatic tissues after upper digestive tract surgery (47). However, Shimizu demonstrated that EOF increased the incidence of postoperative complications in the distal gastrectomy group, especially delayed gastric emptying (27). The possible reason is that EOF may increase food consumption on postoperative day (POD) 4 and thereafter, which led to delayed gastric emptying. A meta-analysis showed that EOF could reduce the incidence of postoperative complications in the total gastrectomy group but increase it in the distal gastrectomy group, possibly due to the small number of RCTs in each subgroup (43). The subgroup analyses in the present meta-analysis showed that EOF was not associated with oral feeding tolerance, readmission rates, or postoperative complications especially anastomotic leakage and pulmonary infection, regardless of whether laparoscopic and open surgery or partial and total gastrectomy or the timing of EOF initiation. Therefore, EOF is safe and feasible for gastric cancer patients after gastrectomy.

The findings of this meta-analysis showed that EOF could significantly shorten the hospital days, accelerate exhaustion, and reduce hospitalization costs. Minig reported that EOF was not only easily absorbed but also could accelerate intestinal peristalsis recovery, protect intestinal mucosal barrier function, and enhance immune response (48). Moreover, intestinal nutrients play an important role in regulating gastrointestinal function (49). Pilichiewicz also demonstrated that dietary macronutrients regulate gastrointestinal motor function and hormone secretion in a load-dependent manner (50). Both Lu and Gao reported that the levels of gastrointestinal hormones were significantly higher on POD 5 in the EOF group, which could speed up the recovery of gastrointestinal function (14, 29). Other studies showed that EOF could be beneficial in leading to faster gastrointestinal function recovery and nutritional improvement of patients with gastric cancer after surgery (51, 52). All these findings provide a theoretical basis for reducing hospital stays and accelerating exhaustion. Shoar revealed that EOF after surgery can promote gastrointestinal function recovery and reduce postoperative hospitalization for patients with upper gastrointestinal malignant tumors (53). Two retrospective studies also indicated that EOF could shorten the length of hospital stay and improve the recovery of bowel functions after the surgery of gastric cancer (14, 45). Two meta-analyses showed that EOF significantly decreased hospital costs for patients with gastric cancer after surgery (41, 43). Sindler also reported that EOF had no risk of several possible postoperative morbidities after upper GI surgeries but has several advantageous effects on a patient’s recovery (54). However, Wang and Hur reported that there were no differences in hospital costs between the two groups, which may be related to the charging standards of different hospitals, and oral on-site nutritional formulations (21, 28). Conversely, the reduction in hospitalization costs by EOF may be related to the shortened length of hospitalization. Subgroup analyses also showed consistent results regardless of whether laparoscopic and open surgery or partial and total gastrectomy or the timing of EOF initiation.

This meta-analysis encompasses a greater number of RCTs and patients compared to prior meta-analyses. Notably, we also analyzed the effect of EOF on postoperative pneumonia, an aspect frequently overlooked in prior meta-analyses. The subgroup analyses further confirm the robustness and reliability of our findings. However, there are still some limitations in our study. First, the inclusion criteria of these RCTs were different; some studies excluded patients with advanced gastric cancer, which contributed to potential heterogeneity in the results. Second, most RCTs had small sample sizes and were single-center studies. Third, the methodological quality was poor due to easy differentiation of feeding methods. Fourth, all RCTs were conducted in Asian countries; thus, the population may not be representative. Fifth, oral feeding regimens were inconsistent, with some patients receiving water or liquid diets and others receiving enteral nutrition preparations. Additionally, the timing of oral feeding varied. Finally, some surgeons and nurses were hesitant to implement early oral feeding in gastric cancer surgery patients, potentially introducing selection bias in the clinical trials and our study. Thus, given these limitations, we conducted subgroup and sensitivity analyses, which yielded consistent results.

## Conclusion

EOF could reduce hospital stays, the time to first flatus, and hospital costs, but it was not associated with oral feeding tolerance, readmission rates, or postoperative complications especially anastomotic leakage and pulmonary infection, regardless of whether laparoscopic or open surgery, partial or total gastrectomy, or the timing of EOF initiation. Therefore, EOF might be safe and effective for gastric cancer patients after gastrectomy. However, further multi-center, multi-regional, and more standardized RCTs are required to address the limitations of the current studies.

## Data Availability

The original contributions presented in the study are included in the article/**Supplementary Material**. Further inquiries can be directed to the corresponding author.

## Supplementary material

Supplementary Figure 1 Subgroup analysis of Hospital day based on operative approach **(A)**, the range of gastrectomy **(B)**, the time to start EOF **(C)**.

Supplementary Figure 2 Subgroup analysis of The time to first flatus based on operative approach **(A)**, the range of gastrectomy **(B)**, the time to start EOF **(C)**.

Supplementary Figure 3 Subgroup analysis of Hospital costs based on operative approach **(A)**, the range of gastrectomy **(B)**, the time to start EOF **(C)**.

Supplementary Figure 4 Subgroup analysis of Oral feeding tolerance based on operative approach **(A)**, the range of gastrectomy **(B)**, the time to start EOF **(C)**.

Supplementary Figure 5 Subgroup analysis of Postoperative complications based on operative approach **(A)**, the range of gastrectomy **(B)**, the time to start EOF **(C)**.

Supplementary Figure 6 Subgroup analysis of Readmission rates based on operative approach **(A)**, the range of gastrectomy **(B)**, the time to start EOF **(C)**.

Supplementary Figure 7 Subgroup analysis of Anastomotic leakage based on operative approach **(A)**, the range of gastrectomy **(B)**, the time to start EOF **(C)**.

Supplementary Figure 8 Subgroup analysis of Pulmonary infection based on operative approach **(A)**, the range of gastrectomy **(B)**, the time to start EOF **(C)**.

Supplementary Figure 9 Funnel plot of Hospital days **(A)**, The time to first flatus **(B)**, Hospital costs **(C)**, Oral feeding tolerance **(D)**, Readmission rates **(E)**, Anastomotic leakage **(F)**, Pulmonary infection **(G)**.
