# Supplementary figures and images for: An updated systematic review and meta-analysis of the efficacy and safety of early oral feeding vs. traditional oral feeding after gastric cancer surgery
Source: Front Oncol. 2024 Sep 4;14:1390065. doi: 10.3389/fonc.2024.1390065 (PMC11408281; doi:10.3389/fonc.2024.1390065)

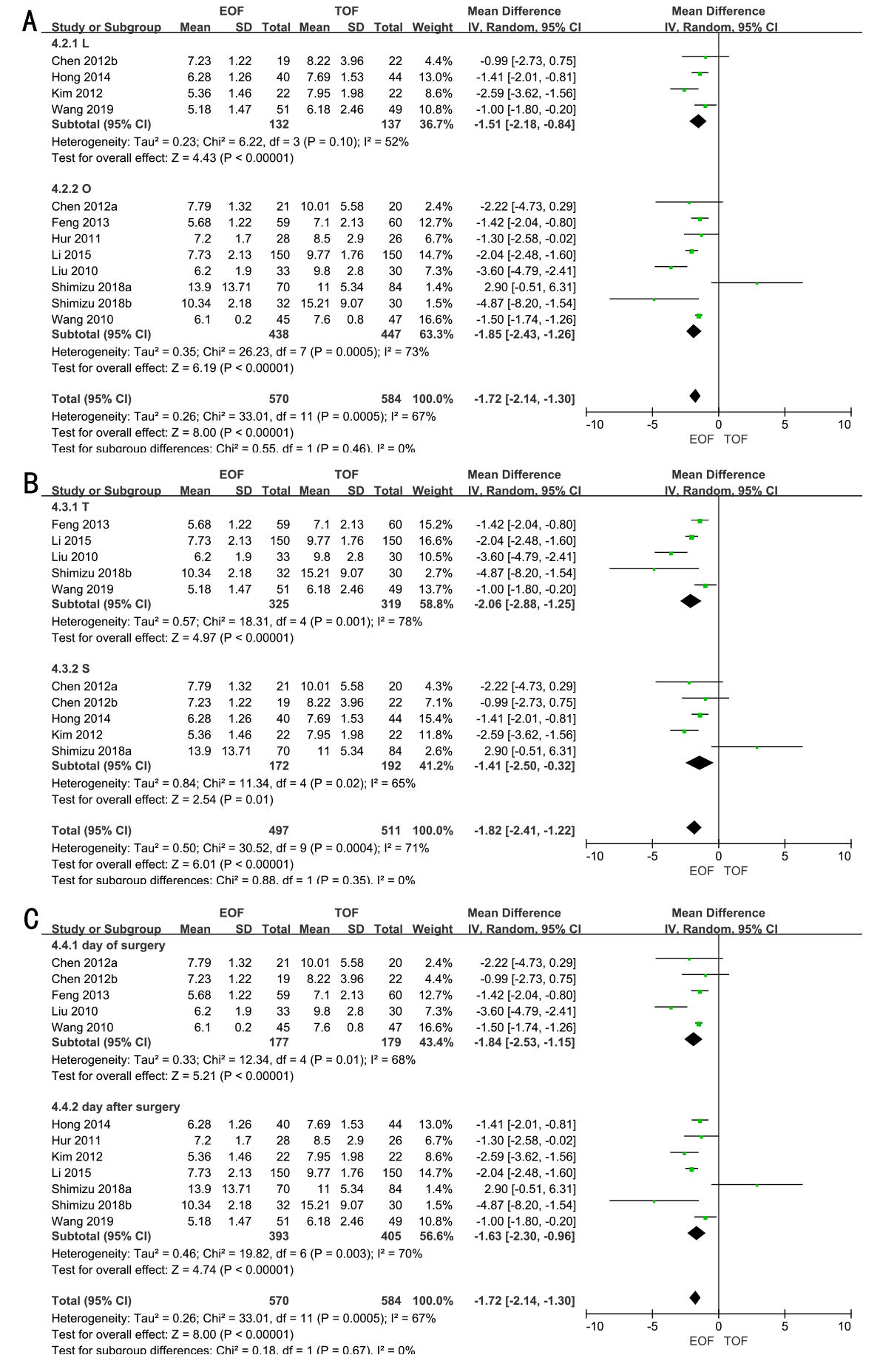

Supplement: Supplementary Figure 1 — Subgroup analysis of Hospital day based on operative approach (A), the range of gastrectomy (B), the time to start EOF (C). [file Image1.tif]

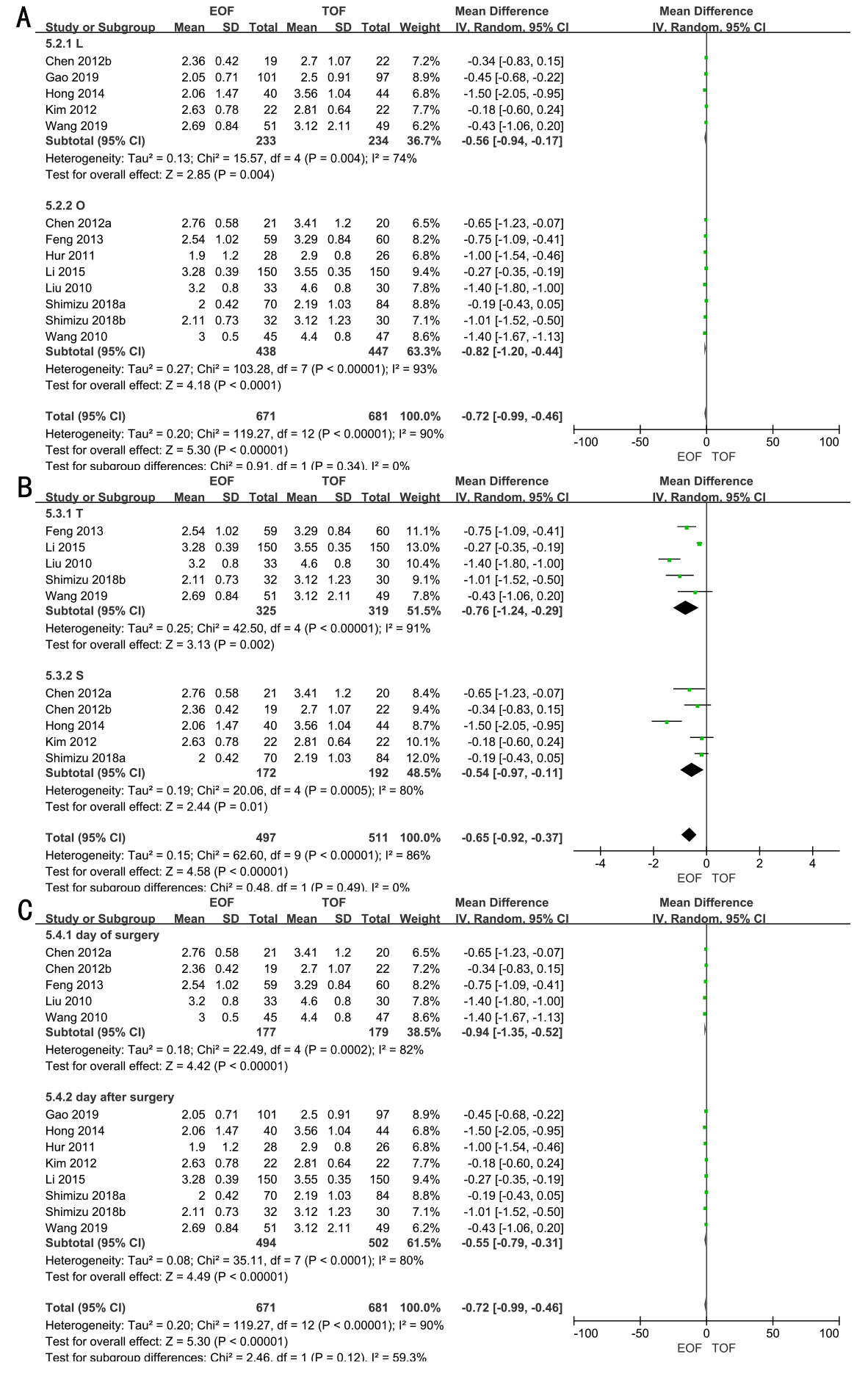

Supplement: Supplementary Figure 2 — Subgroup analysis of The time to first flatus based on operative approach (A), the range of gastrectomy (B), the time to start EOF (C). [file Image2.tif]

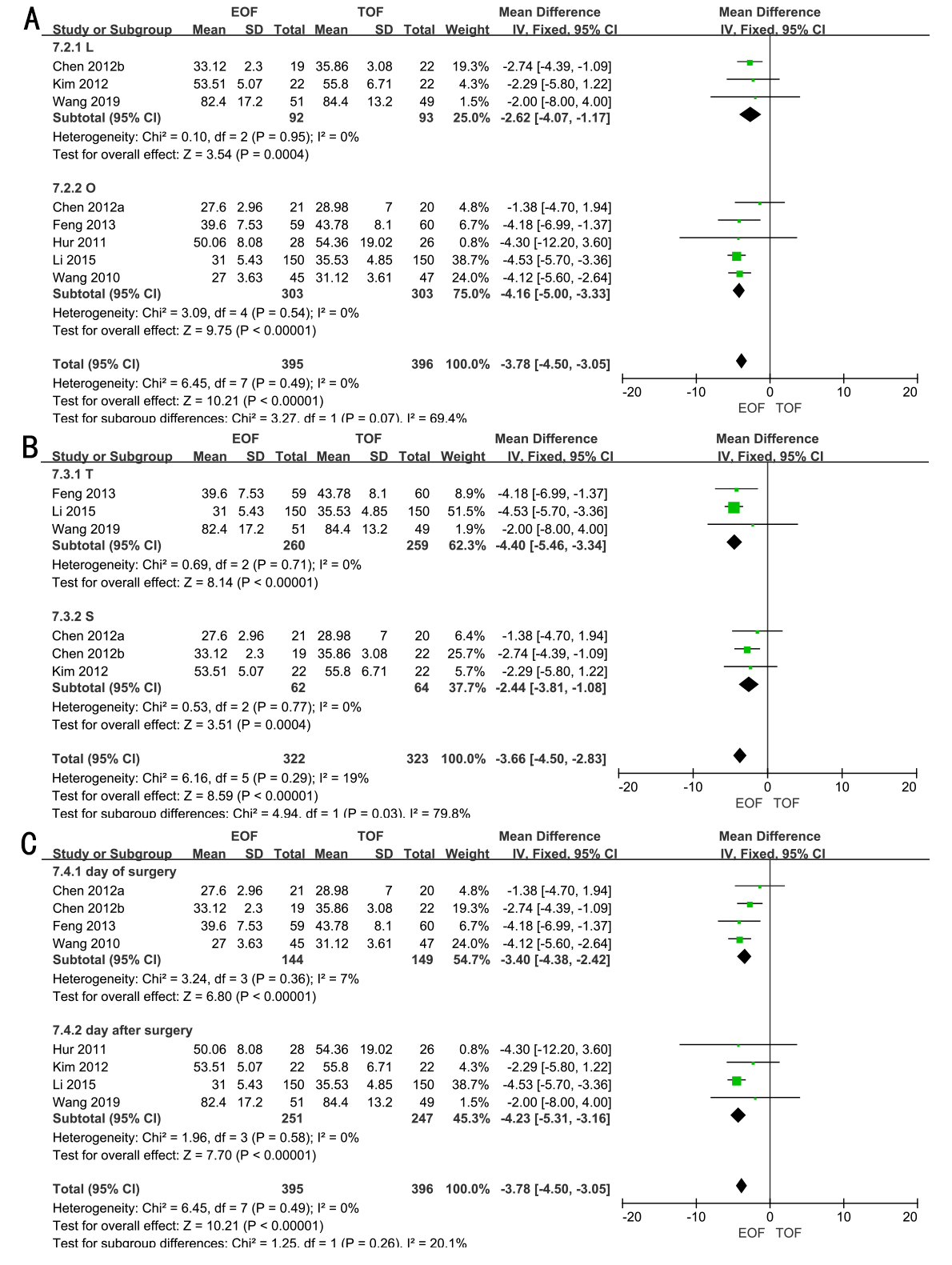

Supplement: Supplementary Figure 3 — Subgroup analysis of Hospital costs based on operative approach (A), the range of gastrectomy (B), the time to start EOF (C). [file Image3.tif]

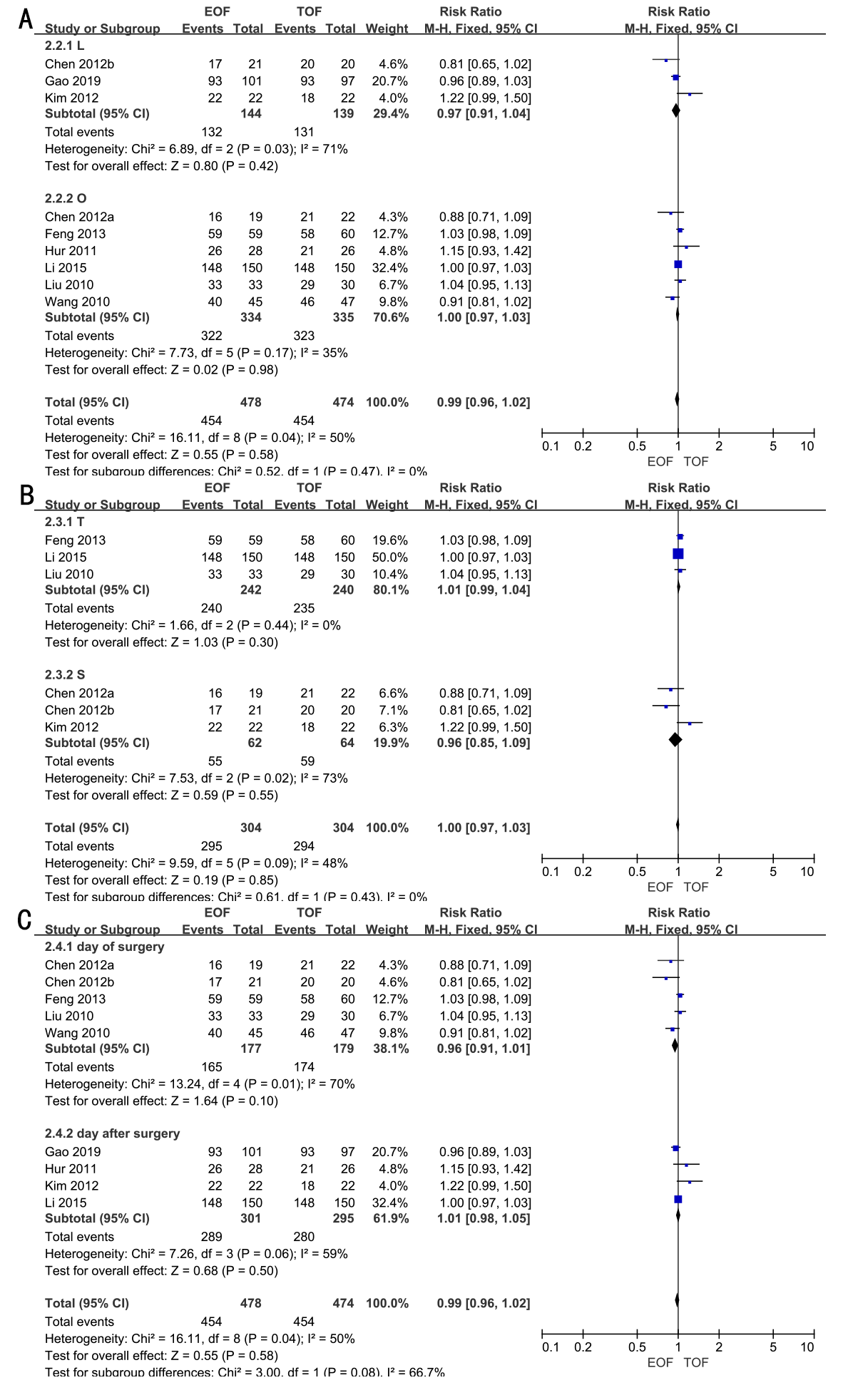

Supplement: Supplementary Figure 4 — Subgroup analysis of Oral feeding tolerance based on operative approach (A), the range of gastrectomy (B), the time to start EOF (C). [file Image4.tif]

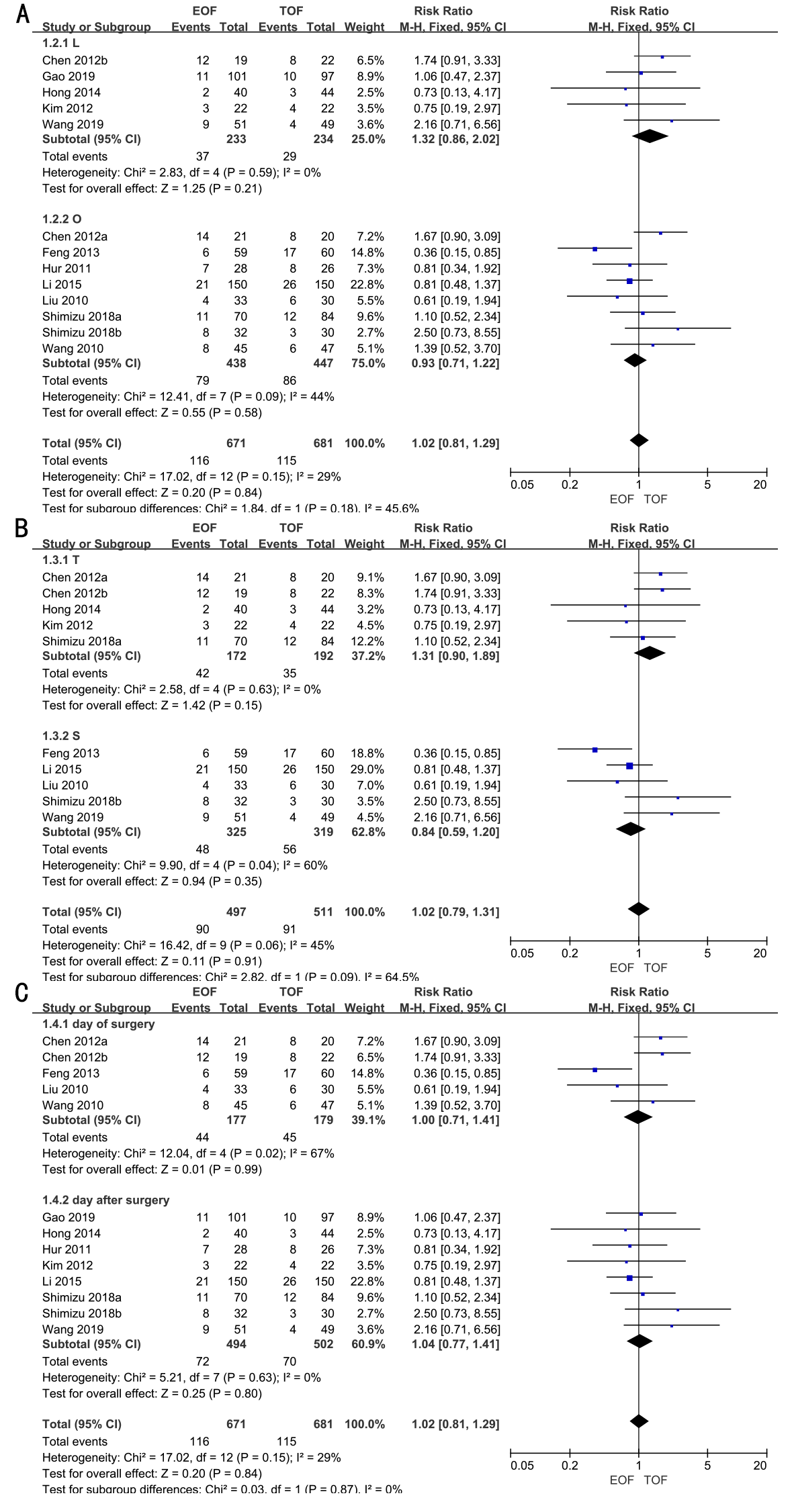

Supplement: Supplementary Figure 5 — Subgroup analysis of Postoperative complications based on operative approach (A), the range of gastrectomy (B), the time to start EOF (C). [file Image5.tif]

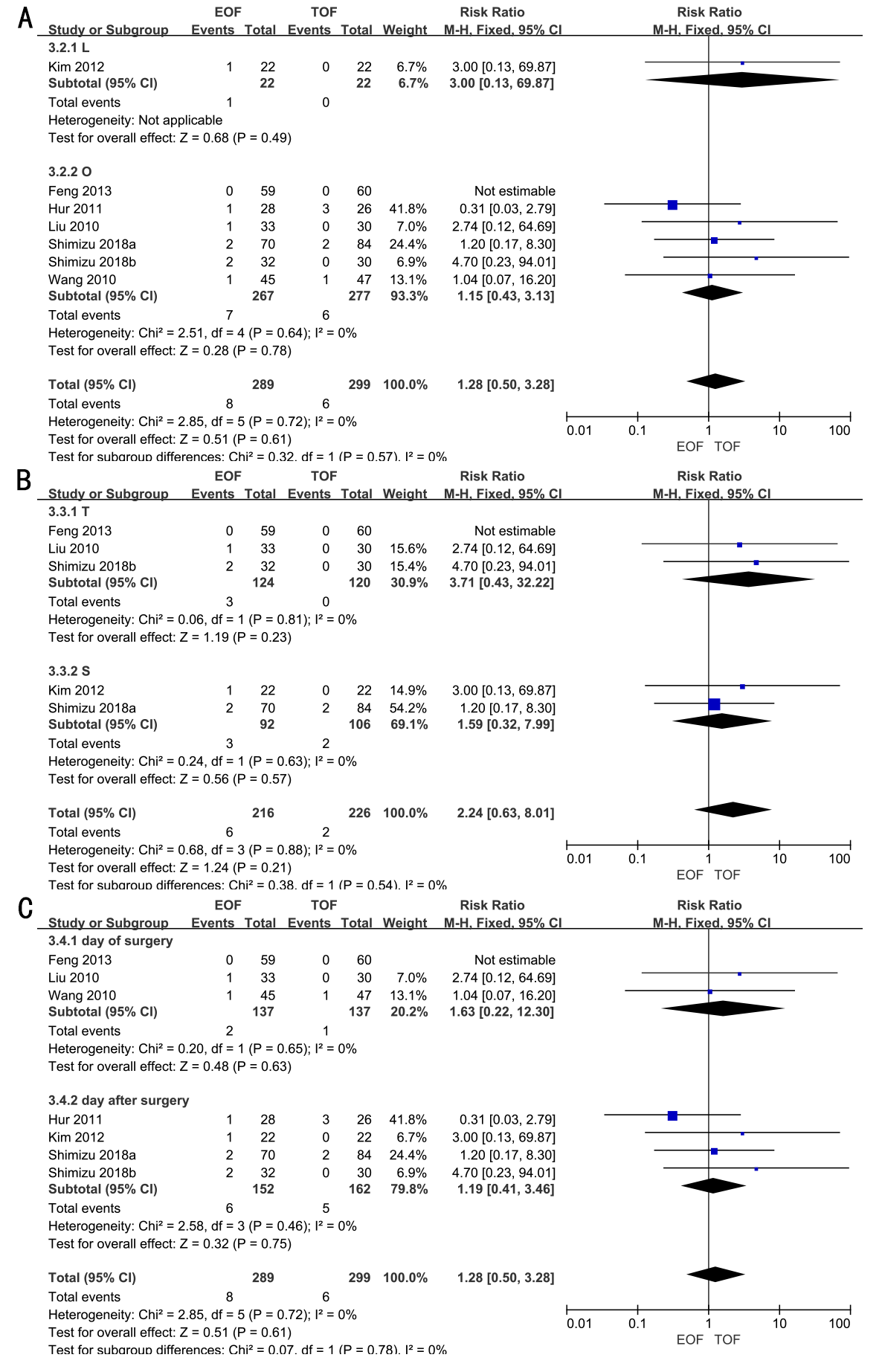

Supplement: Supplementary Figure 6 — Subgroup analysis of Readmission rates based on operative approach (A), the range of gastrectomy (B), the time to start EOF (C). [file Image6.tif]

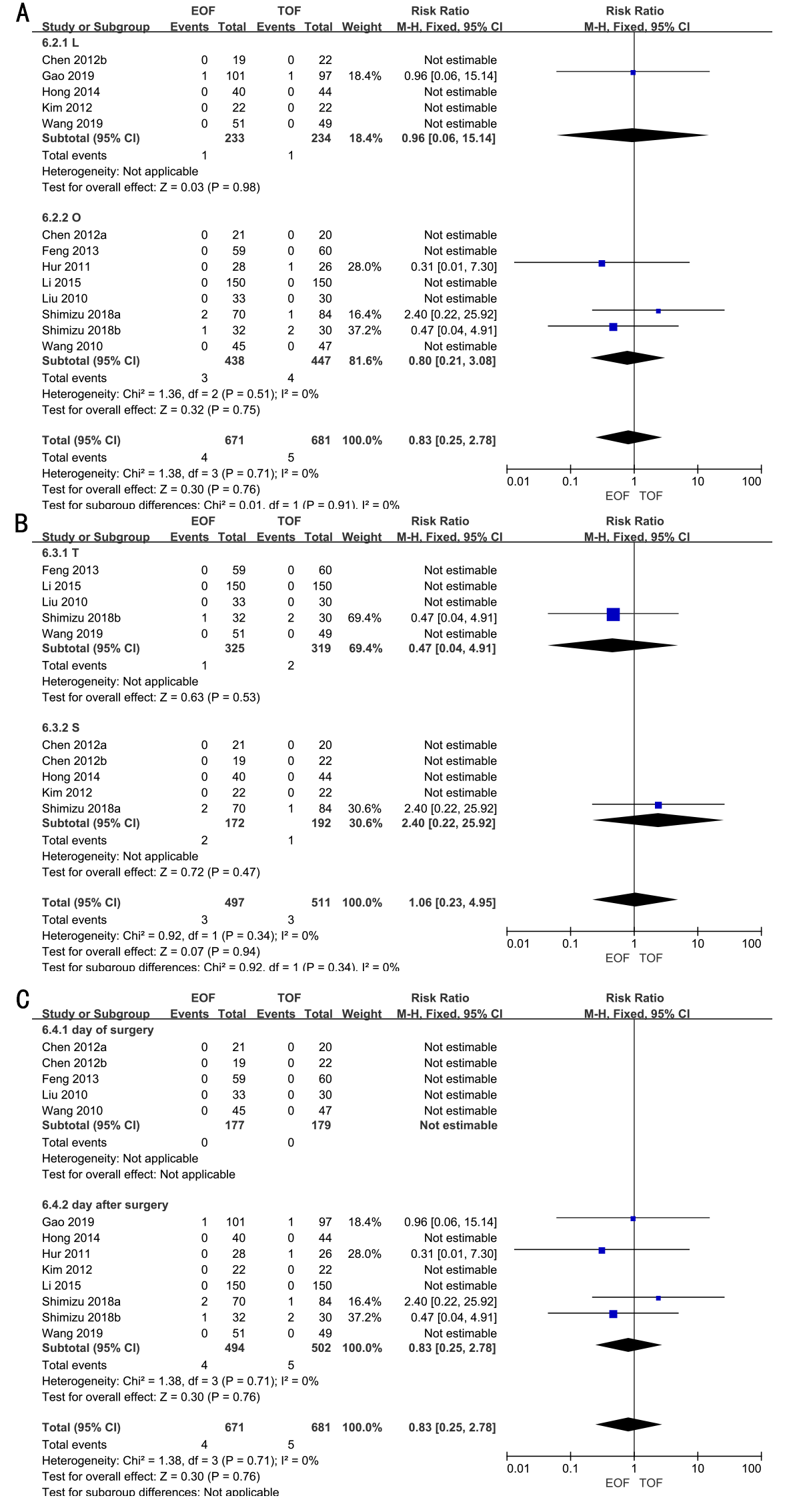

Supplement: Supplementary Figure 7 — Subgroup analysis of Anastomotic leakage based on operative approach (A), the range of gastrectomy (B), the time to start EOF (C). [file Image7.tif]

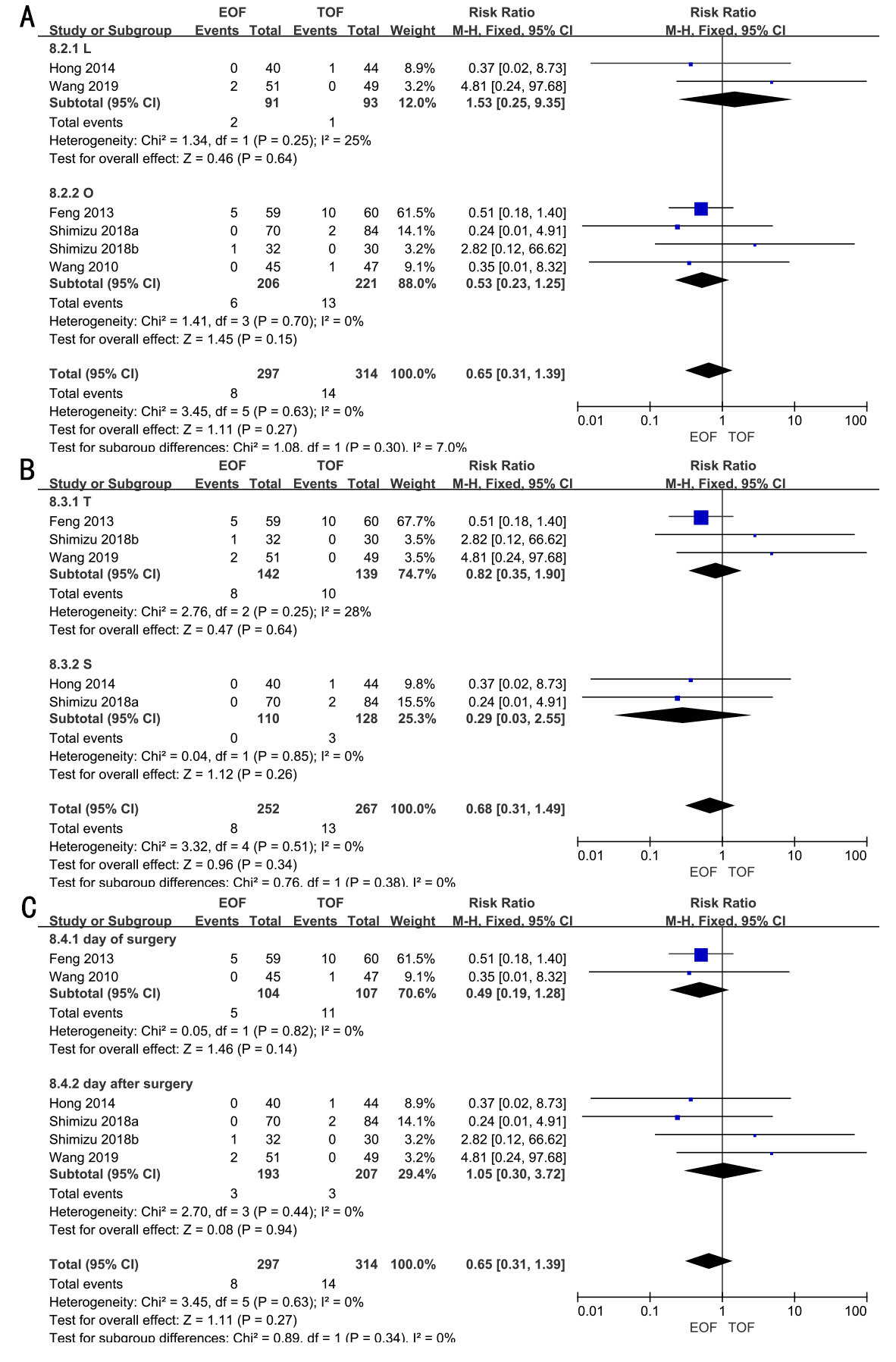

Supplement: Supplementary Figure 8 — Subgroup analysis of Pulmonary infection based on operative approach (A), the range of gastrectomy (B), the time to start EOF (C). [file Image8.tif]

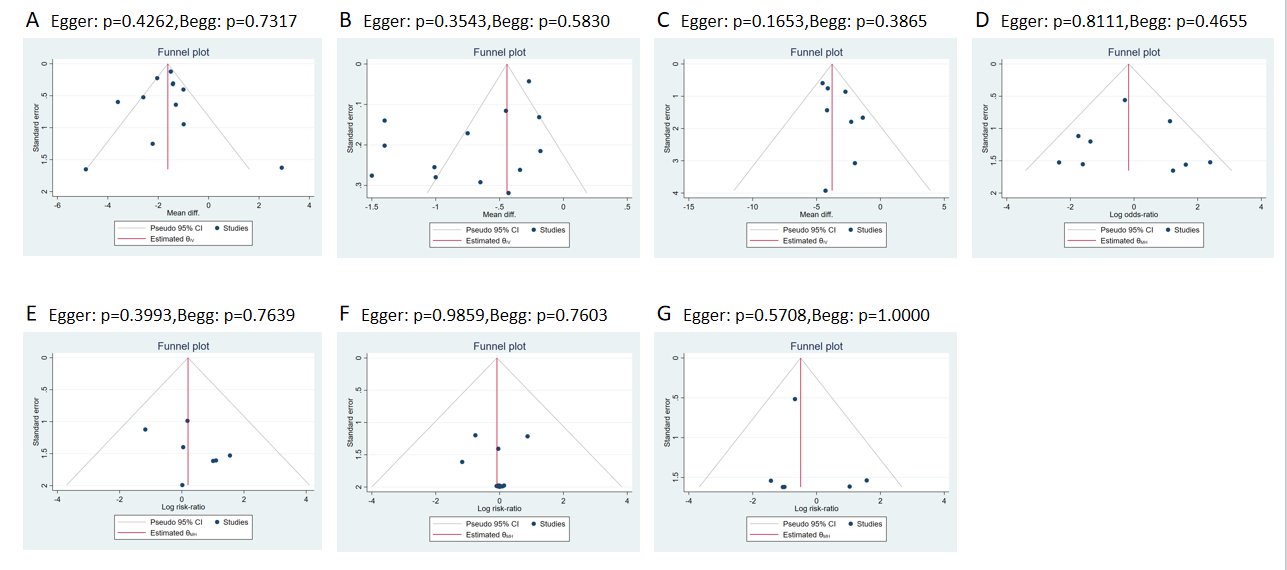

Supplement: Supplementary Figure 9 — Funnel plot of Hospital days (A), The time to first flatus (B), Hospital costs (C), Oral feeding tolerance (D), Readmission rates (E), Anastomotic leakage (F), Pulmonary infection (G). [file Image9.tif]
